# FMCW Radar Human Action Recognition Based on Asymmetric Convolutional Residual Blocks

**DOI:** 10.3390/s24144570

**Published:** 2024-07-15

**Authors:** Yuan Zhang, Haotian Tang, Ye Wu, Bolun Wang, Dalin Yang

**Affiliations:** 1 School of Information Science and Technology, North China University of Technology, Beijing 100144, China; 2022322010106@mail.ncut.edu.cn (H.T.); 2022322010113@mail.ncut.edu.cn (B.W.); yangdalinwk@163.com (D.Y.); 2 Intel Intelligent Edge Computing Joint Research Institute, Nanjing 211100, China; wuye@inchitech.com

**Keywords:** FMCW radar, micro-Doppler, deep learning, attention mechanism, asymmetric convolution, action recognition

## Abstract

Human action recognition based on optical and infrared video data is greatly affected by the environment, and feature extraction in traditional machine learning classification methods is complex; therefore, this paper proposes a method for human action recognition using Frequency Modulated Continuous Wave (FMCW) radar based on an asymmetric convolutional residual network. First, the radar echo data are analyzed and processed to extract the micro-Doppler time domain spectrograms of different actions. Second, a strategy combining asymmetric convolution and the Mish activation function is adopted in the residual block of the ResNet18 network to address the limitations of linear and nonlinear transformations in the residual block for micro-Doppler spectrum recognition. This approach aims to enhance the network’s ability to learn features effectively. Finally, the Improved Convolutional Block Attention Module (ICBAM) is integrated into the residual block to enhance the model’s attention and comprehension of input data. The experimental results demonstrate that the proposed method achieves a high accuracy of 98.28% in action recognition and classification within complex scenes, surpassing classic deep learning approaches. Moreover, this method significantly improves the recognition accuracy for actions with similar micro-Doppler features and demonstrates excellent anti-noise recognition performance.

## 1. Introduction

In recent years, human action recognition has become a research hotspot with a wide range of applications. For example, in the field of medical monitoring [1], human action recognition can provide real-time monitoring, alerting doctors or family members when necessary. In the gaming domain of human–computer interactions [2], human action recognition can be used to respond more intelligently to the player’s actions. In the field of safety and security [3], human action recognition monitors and identifies abnormal behaviors to improve security.

Current methods for realizing activity detection are mainly based on contact and contactless devices. Contact devices primarily obtain information about changes in the human body through sensors carried by the individual and judge movements based on changes in information such as mechanics, angles, and speed [4]. However, the need to carry these devices around affects people’s daily lives, and false alarms may occur due to failure to charge the devices in time.

Contactless devices mainly use optical devices or radar to recognize human movements. Currently, optical-based human action recognition has achieved excellent results in both performance and recognition accuracy. For example, Kerdvibulvech et al. [5,6] proposed a 3D human motion analysis method for reconstruction and recognition. They used 3D gait features computed from 3D human data captured by a projector-camera system. The results show that the biometric recognition technology based on the 3D gait features they proposed shows excellent results on real-world 3D data. The OpenPose model proposed by Cao et al. [7] uses a mathematical model to model a vector field, thereby deriving human posture information based on images captured by an optical camera and outputting human key point data, which can be used for action recognition. However, the data collected by optical equipment depend on the scene lighting. If the light is dim, the optical-based human action recognition system will be seriously affected. Therefore, radar-based human action recognition technology has received increasing attention [8].

Unlike optical sensors, radar sensors are not affected by light or other environmental changes and are extremely sensitive to moving targets. They can accurately measure information such as the distance and speed of the target [9,10]. Additionally, radar sensors can protect personal privacy better than visual sensors and can even perceive through walls [11], making them an ideal choice for practical application scenarios of human action recognition.

In the current literature on radar-based action recognition techniques, action feature extraction and analysis using micro-Doppler spectrograms have become popular research directions. Reference [12] proposed a method to monitor worker activities using the range–Doppler and micro-Doppler characteristics of radar. By carefully analyzing the dynamic characteristics in the radar signal, different types of worker movements can be observed. Arnaoutoglou et al. [13] proposed a low-cost continuous wave radar system to monitor falls in the elderly. They used a short-time Fourier transform to extract effective acceleration from radar signals and calculated indicators such as maximum value, mean value, and variance. Combined with the support vector machine (SVM) [14] algorithm, they achieved a detection accuracy of 95.2%.

In contrast to conventional machine learning techniques like SVM, deep learning methods have shown more significant advantages in identifying and classifying various behaviors. Specifically, deep learning methods achieved higher accuracy and better performance than SVM in the classification task of 12 different behaviors [15]. This advantage mainly stems from the ability of deep learning technology to automatically extract effective features from data without manual intervention or feature engineering.

In the literature [16], Kim and Moon constructed micro-Doppler spectra of seven human body movements using a short-time Fourier transform and employed a convolutional neural network (CNN) for recognition, marking its initial application in this domain. However, the generated micro-Doppler spectrum exhibits significant background noise and lacks clear spectral details, necessitating further enhancement of network recognition accuracy.

It can be seen that many previous studies usually use depth structures or hybrid structures to classify and recognize human actions based on radar micro-Doppler features, but the recognition accuracy of indistinguishable actions is low. In addition, since the production process of radar datasets is usually cumbersome, in real life, researchers often face small-batch radar data classification tasks, and the models are often difficult to fully train or prone to overfitting. Another study [17] utilized a 77 GHz FMCW radar to capture Doppler features from hospital patients and employed a three-layer CNN as a classifier to discern patient behavior. However, the model training in this study primarily relied on data from a single patient, resulting in insufficient diversity within the training set and limited generalization ability. Moreover, since the experiment relies solely on Doppler features, it cannot adequately describe complex actions such as sitting and standing. Additionally, the authors of [18] introduced a convolutional gated recurrent unit neural network (CNN-GRU) that utilizes micro-Doppler spectrograms generated via short-time Fourier transform to classify human body movements of varying durations. However, this study relied on simulated data for experiments, resulting in low model accuracy for specific movements and overlooking the impact of noise interference in real-world environments. Lastly, the authors of [19] introduced a hybrid neural network model to investigate the multi-domain fusion of radar echo data. They derived various spectra by applying a one-dimensional FFT, a two-dimensional FFT, and a short-time Fourier transform to the original radar data. Subsequently, the model employed cross-domain feature fusion to extract more comprehensive features, aiming to enhance recognition accuracy. However, this method still exhibits low recognition accuracy for certain challenging actions.

To address the above problems, this paper proposes a ResNet18 [20] network with an asymmetric convolutional residual block and applies it to human action recognition using FMCW radar. First, a strategy combining asymmetric convolution and the Mish activation function is adopted in the residual block to improve the loss of feature information caused by subtle differences between actions. Second, this paper proposes a lightweight attention mechanism module to make the model pay more attention to the spectral details within the micro-Doppler spectrum while reducing the impact of environmental noise, thereby further improving the robustness and recognition accuracy of the model. Finally, this study used the original radar data [21] released by the University of Glasgow in the UK as the research object. This dataset comprises data gathered from individuals of various genders and ages across diverse locations, covering various actions such as walking, sitting, standing, picking up objects, drinking water, falling, etc. This choice avoids the problem of single micro-Doppler motion data in other studies and provides more diverse and realistic data for research.

The main contributions of this paper are summarized as follows:

- The human activity data collected by millimeter-wave radar were utilized to generate micro-Doppler spectra as features for human activity detection through signal processing algorithms. This approach reduces the complexity of data processing and enhances the privacy and robustness of the system.
- It proposed an asymmetric convolutional residual block to enhance the feature extraction capability of the backbone network without introducing additional hyperparameters, thereby improving the accuracy of action recognition.
- It designed a lightweight attention mechanism module that can effectively improve the network’s representation ability by learning adaptive channel weights while reducing the number of parameters. It is worth noting that it is a plug-and-play module.
- The experiment was verified on a public real-world dataset and achieved excellent accuracy, ensuring the rigor of the experiment.

The rest of this paper is organized as follows. In Section 2, the basic theory of FMCW radar is introduced, and the signal processing method used in this paper to generate the micro-Doppler spectrum map is explained. Section 3 introduces the proposed asymmetric convolutional ResNet18 model for human activity detection in detail. Section 4 compares and analyzes the experimental results. Finally, Section 5 summarizes the work of this paper and outlines future directions for research.

## 2. System Design and Principles

### 2.1. System Composition

**Figure 1:**
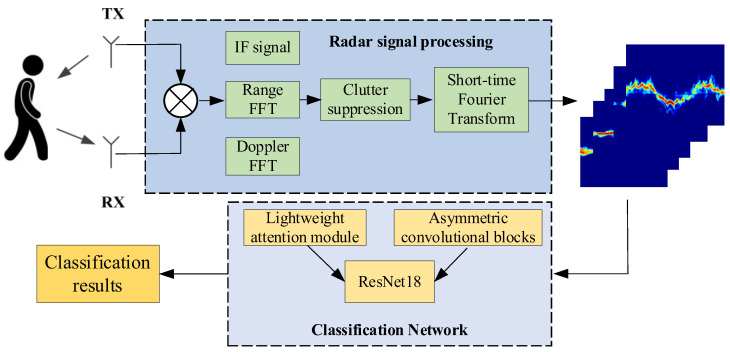
Human action recognition system.

This section introduces the basics of FMCW radar and the principles of extracting target distance and velocity information. It explains the signal processing methods used in generating micro-Doppler spectrograms and illustrates the differences in micro-Doppler spectrograms caused by various activities. The composition of the human activity recognition system designed in this paper is shown in Figure 1.

The radar cube data of the target are collected by radar, and the collected data are processed. By obtaining information such as the distance and speed of the target relative to the radar, a micro-Doppler spectrum diagram is generated. This image is then input into the lightweight ResNet18 neural network proposed in this paper for training to obtain the action classification result.

### 2.2. FMCW Radar Signal Model

**Figure 2:**
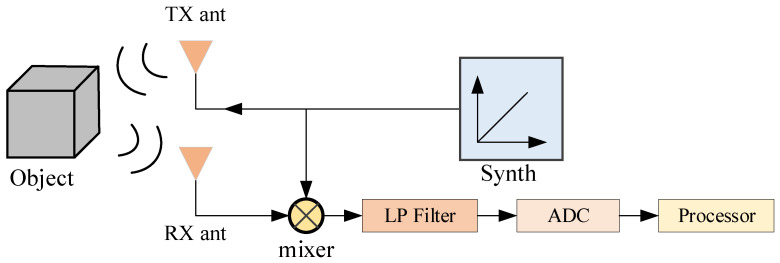
FMCW millimeter-wave radar system schematic block diagram.

**Figure 3:**
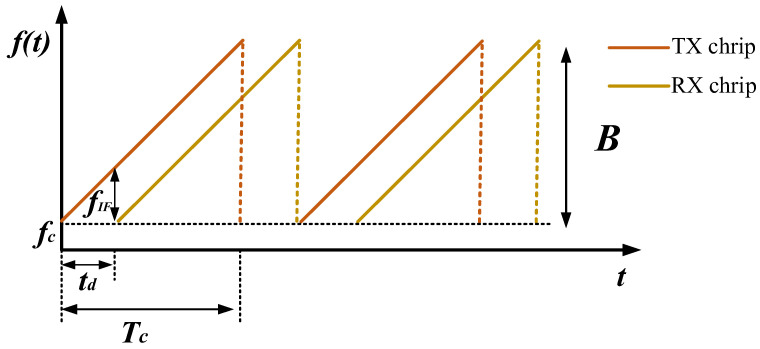
Chirp signal.

The FMCW millimeter-wave radar system comprises the following core components: a transmitter, a receiver, an antenna, and a digital signal processor [22]. During operation, the transmitter converts radio frequency signals into millimeter-wave signals and emits them into space via the antenna. These signals, upon encountering objects, reflect back as radar echoes. The receiver captures and processes these reflected waves by amplifying and filtering the signals. Subsequently, the digital signal processor analyzes these signals to extract crucial data such as the distance and speed of the objects, which are essential for human motion recognition. The schematic block diagram of the FMCW millimeter-wave radar system is illustrated in Figure 2:

This study employs sawtooth wave-modulated FMCW radar to transmit linear frequency modulation signals (chirp signals). The sawtooth shape arises because, during the chirp cycle, the frequency increases linearly from the starting frequency to the ending frequency, creating a gradually rising slope resembling a sawtooth wave. The chirp signals of the transmitting and receiving antennas are depicted in Figure 3.

The chirp signal is generated by the synthesizer and transmitted through the transmitting antenna. The transmitted signal $x_{TX}(t)$ can be represented as:

(1) $$x_{TX}(t)=A_{T}\cos(2\pi f_{c}t+\pi \beta t^{2})$$

In the formula, $A_{T}$ is the transmission power of the radar, $f_{c}$ is the initial frequency of the radar chirp, and $\beta =B/T_{c}$ represents the frequency modulation slope, where $T_{c}$ denotes the duration of the chirp and B denotes the frequency modulation bandwidth. When the radar detects a moving target, the radar’s transmitted signal is reflected back to the receiving antenna via the target. The echo signal $x_{RX}(t)$ received from the target can be expressed as:

(2) $$x_{RX}(t)=A_{R}\cos(2\pi f_{c}(t-t_{d})+\pi \beta {\left(t-t_{d}\right)}^{2})$$

In the formula, $t_{d}=2R(t)/c$ denotes the time delay for the radar antenna to receive the return signal echo, and R(t) denotes the distance between the radar-detected target and the return signal echo.

The mixer mixes the transmitted signal and the received signal and then filters out the high-frequency components through a low-pass filter to generate an intermediate-frequency signal. The intermediate-frequency signal $x_{IF}(t)$ can be represented as:

(3) $$x_{IF}(t)=\frac{1}{2}A_{T}A_{R}\cos(2\pi \beta (t_{d}t-\frac{1}{2}{t^{2}}_{d})+2\pi f_{c}t_{d})$$

Since $t_{d}$ is very small in actual measurements, ${t^{2}}_{d}$ can be assumed to be zero. Therefore, the frequency of the intermediate-frequency (IF) signal can be represented as follows:

(4) $$f_{IF}=\beta t_{d}=\frac{B}{T_{c}}\cdot \frac{2R}{c}$$

When the radar detects multiple targets, the Fourier transform of the intermediate-frequency–time domain signal can separate different frequencies $f_{IF}$ and their corresponding amplitudes. The distance of the target relative to the radar can be expressed as:

(5) $$R=\frac{cT_{c}f_{IF}}{2B}$$

FMCW millimeter-wave radar calculates the target speed through the phase difference of the intermediate-frequency signal. When the moving target has a radial motion speed v relative to the radar and the interval time of sending the Chirp signal is $T_{c}$, the phase difference of the intermediate-frequency signal can be expressed as:

(6) $$\Delta \varphi =\varphi_{2}-\varphi_{1}=\frac{4\pi vT_{c}}{\lambda }$$

From the above formula, we can determine that the radial motion speed of the target relative to the radar can be expressed as:

(7) $$v=\frac{\Delta \varphi \lambda }{4\pi T_{c}}$$

Through the aforementioned processing steps, the characteristics of distance–time and distance–speed of the target can be described.

### 2.3. Micro-Doppler Spectrum Preprocessing

The previous section covered the basic information of FMCW radar, along with the theory for extracting target distance and velocity information. This section will elaborate on the signal processing methods used in this paper to generate micro-Doppler spectrograms.

**Figure 4:**
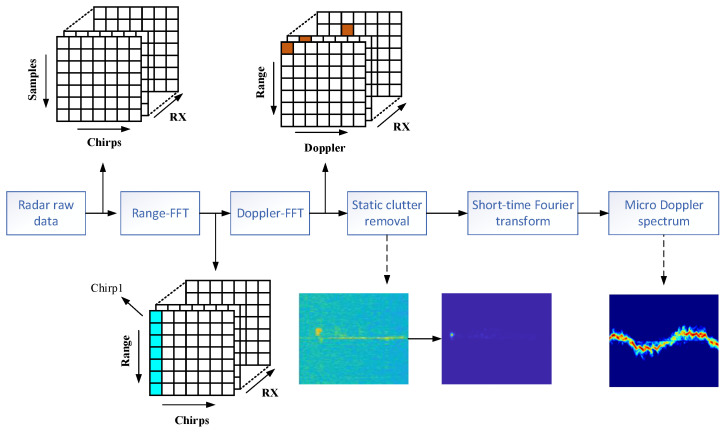
Micro-Doppler spectrogram construction flow chart.

The echo data collected by the FMCW radar are stored in binary form and rearranged into a multidimensional matrix. The process of constructing a micro-Doppler spectrum based on the radar raw data in this paper is shown in Figure 4, where each column in the matrix represents an intermediate-frequency chirp and each point is a sampling point. Since the interval between sampling points in each intermediate-frequency chirp is much smaller than the interval between chirps, the vertical axis of the matrix is usually called the fast time dimension, and the horizontal axis is called the slow time dimension.

First, this paper performs a fast Fourier transform (FFT) on the radar data in the fast time dimension to transform the matrix into distance units. Then, a Doppler FFT is performed on the distance unit where the target is located in the slow time dimension to obtain the range–Doppler map.

However, in the radar data collection environment, the radar data contain a large number of static objects or irrelevant targets, collectively referred to as clutter, in addition to detecting human motion. These radar echoes accumulate at the zero Doppler position, thereby interfering with the generation of subsequent micro-Doppler spectra. This interference may affect the effectiveness of deep learning algorithms in image classification tasks. To address this issue, this study employs Moving Target Indicator (MTI) technology to suppress clutter, aiming to enhance the radar signal-to-noise ratio and improve the accuracy of moving target detection [23].

The core concept of MTI technology revolves around the observation that the phase of stationary targets remains unchanged across successive radar pulses, whereas the phase of moving targets varies. By subtracting the return signals of two consecutive radar pulses, phases that remain identical (indicating stationary targets) are suppressed, while changing phases (indicating moving targets) are preserved. This method effectively filters out stationary objects, allowing the radar system to focus on detecting moving targets.

**Figure 5:**
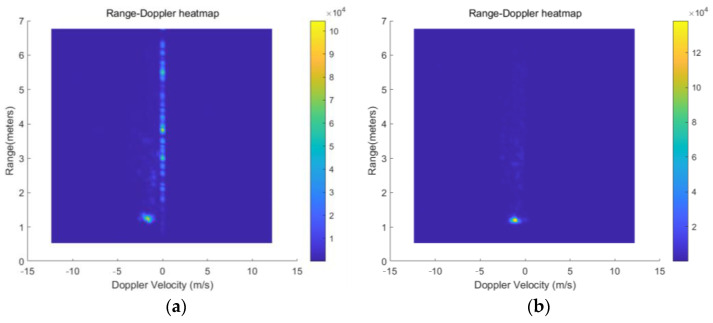

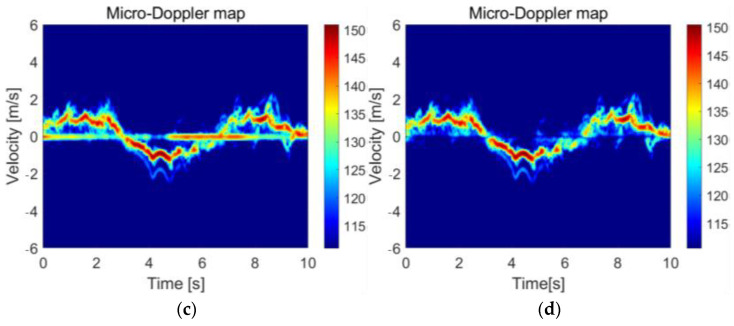
Range–Doppler heatmap: (**a**) before MTI and (**b**) after MTI. Micro-Doppler spectrogram: (**c**) before MTI and (**d**) after MTI.

The range–Doppler and micro-Doppler spectrum diagrams before and after the adoption of MTI are shown in Figure 5a–d. The DC component of the signal has been well suppressed. This step significantly improves the quality of the micro-Doppler spectrogram.

The time domain spectrum information of the micro-Doppler signal can be effectively extracted using time–frequency analysis technology, which concurrently analyzes the time and frequency characteristics of the signal. This process generates a micro-Doppler spectrum diagram that reflects the motion characteristics of the target. To extract the micro-Doppler characteristics of the target, this experiment utilizes the Short-Time Fourier Transform (STFT) to produce the micro-Doppler spectrum image from the target’s echo signal.

The STFT divides the time-varying signal into segments of equal length and applies the Fourier transform to each segment. The mathematical representation of the STFT is as follows:

(8) $$STFT\left(t,f\right)=\int_{-\infty }^{+\infty }x(\tau )w(\tau -t)e^{-j2\pi f\tau }d\tau$$

**Figure 6:**
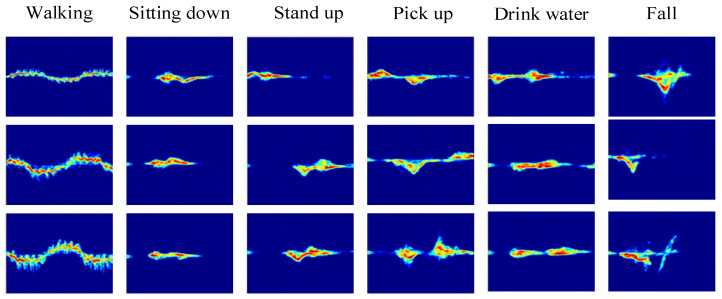
Micro-Doppler spectra of six actions.

In the formula, w(τ) represents the window function, and this paper employs the Hamming window. Figure 6 illustrates the micro-Doppler spectra of six different human body movements obtained after radar signal preprocessing. Each row in the micro-Doppler spectrum diagram represents actions performed by different individuals. These spectra capture the subtle movements of the target through oscillation. Due to variations in the speed of different body parts during various actions, micro-Doppler spectra can effectively be utilized for human action recognition.

## 3. Micro-Doppler Spectrogram Action Recognition Model

### 3.1. ResNet18 Backbone Network

**Figure 7:**
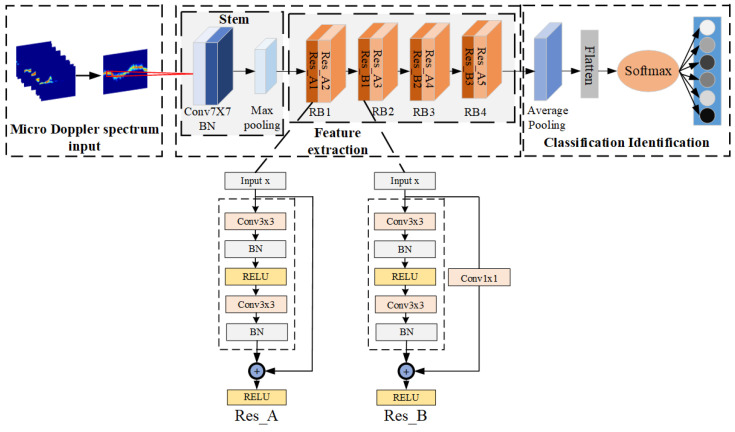
Resnet18 backbone network.

To balance high accuracy and a low model parameter count in micro-Doppler spectrum classification, this paper selects ResNet18 as the backbone network. ResNet18 is chosen for its moderate number of network layers and the incorporation of a residual structure, effectively addressing the issue of information loss as the neural network depth increases. The structure of the ResNet18 network is depicted in Figure 7. The network primarily consists of a 7 × 7 large convolutional layer and a maximum pooling layer, followed by feature extraction using four residual blocks (RBs). Each residual block includes convolutional layers, batch normalization layers, and ReLU activation functions. Finally, the output is obtained through global average pooling and fully connected layers.

The enhanced ResNet18 proposed in this study, incorporating asymmetric convolutional residual blocks, consists of three main components:

1. The Stem input structure at the beginning of the network is modified as follows: the original 7 × 7 large convolution kernel is replaced by three consecutive 3 × 3 small convolution kernels in series. Additionally, a ReLU activation function is added after each 3 × 3 convolution layer. This modification aims to enhance the nonlinear capabilities of the network, enabling it to better capture intricate patterns and features in the input data;
2. The strategy adopted involves fusing asymmetric convolutional layers with Mish activation functions to replace standard 3 × 3 convolutional layers. This approach utilizes convolution kernels of different sizes (1 × 3, 3 × 1, and 3 × 3) in parallel. By doing so, subsequent layers of the network can gather more diverse and richer information from the input data. Importantly, this is achieved while maintaining a relatively low computational cost, enhancing the network’s learning ability and its capacity to extract meaningful features from complex datasets such as micro-Doppler spectra;
3. Improved channel attention and spatial attention modules are added to the residual block to enhance the network’s focus on the action area of the micro-Doppler map. This enhancement allows the model to better learn important features within the map.

### 3.2. ResNet18 Initial Stem Module Convolution Kernel Replacement

The initial part of ResNet18 includes a large convolutional layer with a 7 × 7 kernel size and a stride of 2, followed by a 3 × 3 maximum pooling layer with a stride of 2. This section, known as the Stem module, is crucial for extracting preliminary image features. These features are subsequently refined and combined in later layers, gradually reducing the input image resolution to optimize computational efficiency.

**Figure 8:**
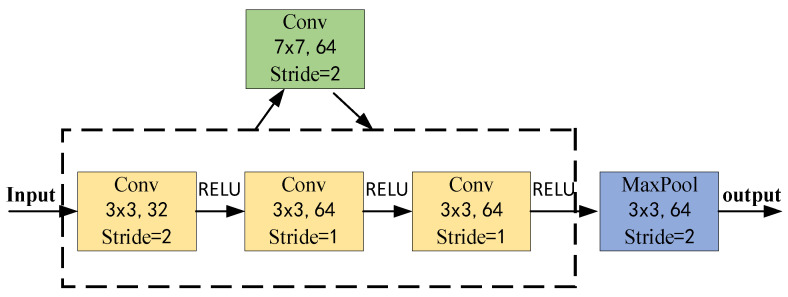
Stem module 7 × 7 large convolution kernel replacement.

In this study, the Stem module is enhanced by replacing the original 7 × 7 convolutional layer with three consecutive 3 × 3 convolutional layers. Additionally, a ReLU activation function is applied after each convolutional layer. This modification aims to improve the network’s ability to effectively capture detailed image features. The improved block diagram is depicted in Figure 8.

The calculation formula for the receptive field is as follows:

(9) $$F_{i}=(F_{i-1}-1)\times Stride+K_{size}$$

where $F_{i}$ represents the receptive field of the layer i; Stride represents the step size of the layer i; and $K_{size}$ represents the convolution kernel size, also known as the filter size.

Given that the receptive field size of the initial layer in the network is 1, the receptive field size of the large convolutional layer, which features a 7 × 7 kernel size and a stride of 2, would be as follows:

(10) $$F_{1}=(1-1)\times 2+7=7$$

The receptive field of a 3 × 3 small convolutional layer with a stride of 2 and two consecutive 3 × 3 small convolutional layers each with a stride of 1 in series is as follows:

(11) $$F_{1}=(1-1)\times 2+3=3$$

(12) $$F_{2}=(3-1)\times 1+3=5$$

(13) $$F_{3}=(5-1)\times 1+3=7$$

It can be seen from the formula that the size of the receptive field remains unchanged. Through this design, the network can increase depth and nonlinearity without losing coverage of the input information.

### 3.3. Convolutional Attention Module

**Figure 9:**
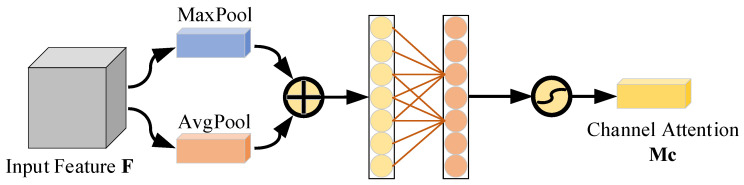
ICBAM’s channel attention module.

Radar data collected in various complex scenarios often include noise, which can adversely affect model recognition. Introducing the Convolutional Block Attention Module (CBAM) [24] can effectively suppress complex background interference and extract key pixel areas. The CBAM integrates both the Channel Attention Module (CAM) and the Spatial Attention Module (SAM). The channel attention module primarily addresses variations in the micro-Doppler spectrum by utilizing a multi-layer perceptron with two fully connected layers to process information within the feature channel dimension. However, scaling coefficients used to reduce and then increase the dimension of feature channels can disrupt the direct correspondence between feature channels and their weights. This process may lead to the loss of some feature information during dimensionality reduction. To address these issues, this paper proposes using a one-dimensional convolution instead of a multi-layer perceptron structure in the channel attention mechanism. This change not only maintains direct correspondence between feature channels and their weights but also significantly reduces the number of parameters. Figure 9 illustrates the channel attention module of the ICBAM. The process is as follows: the input feature map *F* undergoes global average pooling and global maximum pooling in the spatial dimension. These pooled results are then fused across the channel dimension to obtain $F_{\mathrm{add}}$. Subsequently, a one-dimensional convolution with a kernel length of *k* is used to process the feature information across *k* adjacent channels in $F_{\mathrm{add}}$. Finally, the value is normalized to the range of 0 to 1 using the Sigmoid activation function to generate the channel attention weight $M_{C}$, which is calculated as follows:

(14) $$\begin{array}{l} M_{C}(F)=\sigma (f_{1D}^{k}(AvgPool(F)+MaxPool(F))) \end{array}$$

where $f_{1D}^{k}$ represents a one-dimensional convolution operation with a convolution kernel size of *k*. The size of the one-dimensional convolution kernel *k* can be adaptively determined according to the number of channels of the input feature map. The formula is as follows:

(15) $$k={\left|\frac{lbC}{2}+\frac{1}{2}\right|}_{odd}$$

where ${\left|a\right|}_{odd}$ represents the closest odd number to ‘a’ upward.

The spatial attention module focuses on the positional information of pixels in the micro-Doppler spectrum, and the size of its convolution kernel determines its feature extraction capability. Larger-scale convolution kernels encompass a broader receptive field, facilitating enhanced aggregation of spatial context information. Conversely, smaller-scale convolution kernels excel at extracting local subtle features. Therefore, relying solely on a single scale of convolution kernels may result in incomplete feature extraction.

To address this issue, this paper proposes using a combination of a 1 × 1 convolutional layer and three 3 × 3 dilated convolutional layers, each with dilation factors of 1, 2, and 3, respectively. This approach aims to capture multi-scale contextual information. The outputs from these multi-scale convolutions are concatenated and fused, followed by a 1 × 1 convolutional layer to reduce the channel dimension.

**Figure 10:**
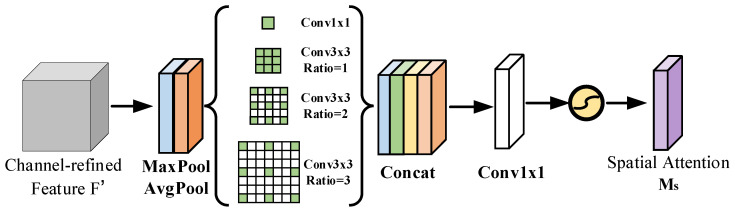
ICBAM’s spatial attention module.

Finally, the value is normalized to the range of 0 to 1 through the Sigmoid activation function to generate the spatial attention weight $M_{S}$. The spatial attention module of the ICBAM is shown in Figure 10. Its calculation formula is as follows:

(16) $$\Lambda (\cdot )=[Conv_{\left(1,1\right)}(\cdot ),Conv_{\left(3,1\right)}(\cdot ),Conv_{\left(3,2\right)}(\cdot ),Conv_{\left(3,3\right)}(\cdot )]$$

(17) $$M_{S}(F^{\prime })=\sigma (f^{1\times 1}(concat(\Lambda [AvgPool(F^{\prime });MaxPool(F^{\prime })])))$$

where Λ is a multi-scale convolution path; $Conv_{\left(i,j\right)}$ represents a convolution operation with a dilated convolution kernel whose original size is *i* and whose dilation factor is *j*; and $f^{1\times 1}$ represents a convolution operation with a 1 × 1 convolution kernel size.

### 3.4. Asymmetric Convolutional Residual Block

The residual block in the ResNet18 network utilizes a 3 × 3 convolutional kernel to deeply explore high-level representations of input features, enhancing the model’s ability to capture features across different scales through nonlinear transformations with the ReLU activation function. However, in identifying specific actions in the micro-Doppler spectrum, certain action forms and directions exhibit highly similar characteristics, leading to identification confusion. To address the challenge of subtle feature differences between actions, this paper proposes an asymmetric convolutional residual block aimed at distinguishing fine-grained micro-Doppler spectra [25].

**Figure 11:**
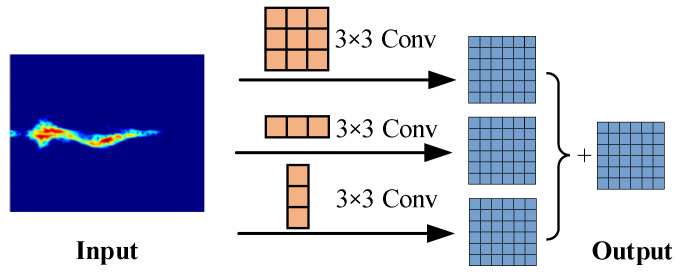
Asymmetric convolution.

This method replaces each 3 × 3 convolutional kernel in the residual block with a sequence of three convolutional layers: 3 × 3, 1 × 3, and 3 × 1 convolutions, optimized with batch normalization (BN) during training. Subsequently, the outputs from these three convolutional layers are combined to produce the output of the convolutional layer, continuing the forward propagation. Because this process incorporates asymmetric operations like 1 × 3 and 3 × 1 convolutions, it is termed asymmetric convolution, as illustrated in Figure 11.

Compared to the conventional convolutional backbone network, the use of asymmetric convolution can be achieved in the inference stage and does not increase the computation time. In addition, it can obtain richer feature information, and the final inference results will be more accurate. The formula for convolutional fusion is as follows:

(18) $$F^{\prime (j)}=\frac{\theta_{j}}{\delta_{j}}F^{\left(j\right)}⊕\frac{{\bar{\theta }}_{j}}{{\bar{\delta }}_{j}}{\bar{F}}^{\left(j\right)}⊕\frac{{\overset{⌢}{\theta }}_{j}}{{\overset{⌢}{\delta }}_{j}}{\overset{⌢}{F}}^{\left(j\right)}$$

(19) $$b_{j}=-\frac{\mu_{j}\theta_{j}}{\delta_{j}}-\frac{{\bar{\mu }}_{j}{\bar{\theta }}_{j}}{{\bar{\delta }}_{j}}-\frac{{\overset{⌢}{\mu }}_{j}{\overset{⌢}{\theta }}_{j}}{{\overset{⌢}{\delta }}_{j}}+\beta_{j}+{\bar{\beta }}_{j}+{\overset{⌢}{\beta }}_{j}$$

(20) $$O_{:\;:k}=\sum_{k=1}^{c}N_{:\;:k}*F_{:\;:k}^{\prime (j)}+b_{j}$$

where $F^{\prime (j)}$ and $b_{j}$ are the convolution kernel and bias of the fused standard convolution, respectively, and O is the final output of the fused convolutional kernel. $\theta_{j}$, ${\bar{\theta }}_{j}$, and ${\overset{⌢}{\theta }}_{j}$ are the weights of the 3-branch convolution kernel. $\delta_{j}$, ${\bar{\delta }}_{j}$, and ${\overset{⌢}{\delta }}_{j}$ are the variances of the 3-branch convolution kernel. $\mu_{j}$, ${\bar{\mu }}_{j}$, and ${\overset{⌢}{\mu }}_{j}$ are the batch normalized means of the 3-branch convolution kernel. $\beta_{j}$, ${\bar{\beta }}_{j}$, and ${\overset{⌢}{\beta }}_{j}$ are 3-branch convolution kernel biases.

In addition, the activation function is modified. The activation function introduces nonlinearities into the network model, which effectively prevents gradient vanishing and explosion and enhances the neural network’s expressive capacity, thereby improving the final classification performance. The ReLU activation function can be defined as follows:

(21) $$ReLU(x)=\max(0,x)\{\begin{array}{c} x,x\geq 0\\ 0,x<0 \end{array}$$

It can be seen from the formula that when the value of the input vector is less than 0, the function will always remain 0. This results in corresponding parameters not being updated, which may cause the loss of part of the information in the micro-Doppler spectrum and ultimately affect recognition accuracy. To solve this problem, this paper adopts a smoother non-monotonic function: the Mish activation function is utilized to replace the ReLU activation function in the original residual block. The expression of the Mish activation function is as follows:

(22) $$Mish(x)=x\cdot \tanh(\ln(1+e^{x}))$$

**Figure 12:**
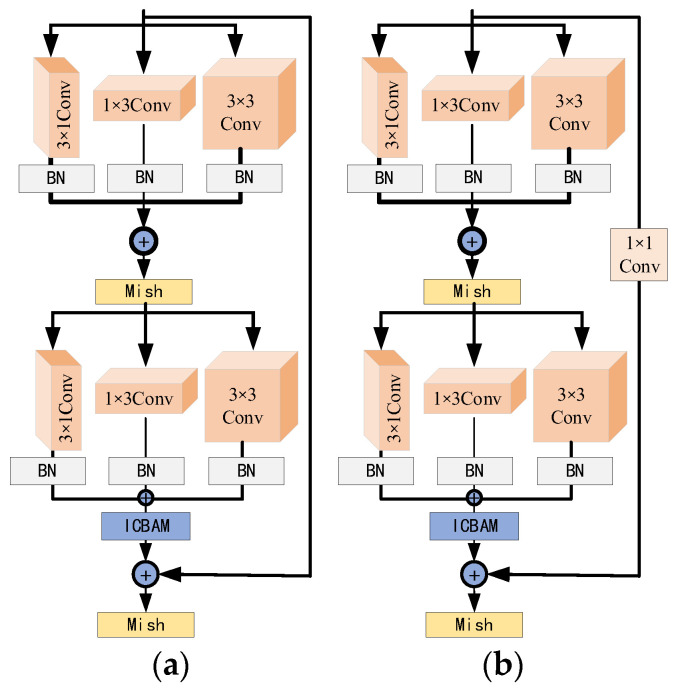
Two types of residual blocks: (**a**) Res_A-ICBAM and (**b**) Res_B-ICBAM.

The Mish activation function combines the properties of the hyperbolic tangent and softplus functions [26]. It retains the sparsity advantage of the ReLU activation function on the positive semi-axis and improves the processing of negative semi-axis values, effectively avoiding the gradient vanishing problem. These features not only enhance the nonlinear performance of the model but also improve the accuracy and stability of gradient propagation, thereby optimizing the overall network performance. The ICBAM is embedded into the residual block, and the improved asymmetric convolutional residual block is shown in Figure 12.

**Figure 13:**
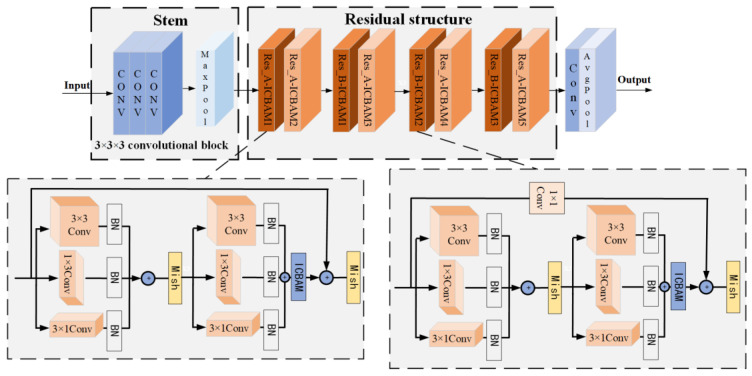
ResNet18-ICBMA-AC network structure.

Based on the above improvements, the final micro-Doppler spectrum action recognition network structure is shown in Figure 13 and is named ResNet18-ICBAM-AC.

## 4. Experimental Verification and Comparative Analysis

### 4.1. Dataset Introduction

The public data used in this experiment originated from the original radar dataset of the University of Glasgow, UK, collected by Ancortek’s FMCW radar. This radar operates in the 5.8 GHz frequency band with a bandwidth of 400 MHz, and its chirp sweep time is 1 millisecond. A total of 56 subjects participated in the experiment, exhibiting diverse individual characteristics such as age and height.

**Figure 14:**
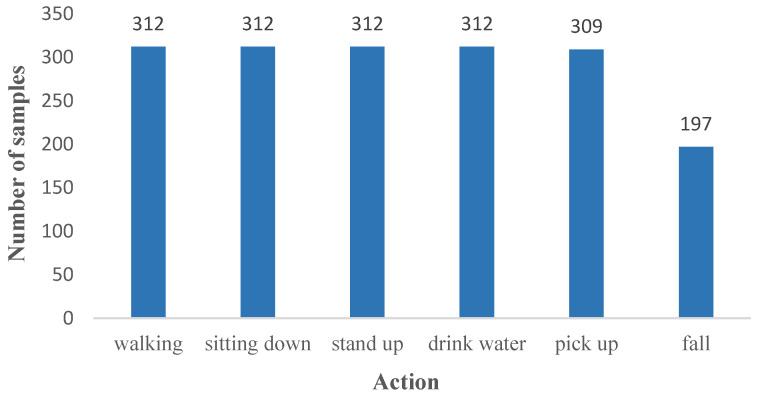
Sample graph of each action in the dataset.

Unlike previous radar data collections conducted mainly in indoor laboratory settings, this dataset covers a broader range of environments, including open areas and laboratories. The data collection took place across seven different venues, each contributing varying numbers of samples for each action, as depicted in Figure 14.

### 4.2. Model Parameter Settings

The environmental parameters of this experiment are shown in Table 1.

The total number of micro-Doppler datasets generated after signal processing was 1754. Due to inconsistent image sizes in the dataset, all feature maps were resized to 224 × 224 pixels before being input into the network. The pixels were then normalized to 0 to 1, followed by standardization with a mean of 0.5 and a standard deviation of 0.5. This method ensures uniform scales across different features, thereby enhancing model training efficiency.

To mitigate the potential effects of image order on the model’s learning effectiveness during training, a strategy of randomly shuffling the training set images was implemented. This approach improves the model’s generalization ability to unseen data.

In addition, the model employs a cross-entropy loss function to compute the error between the predicted output and the true label values. The gradient is then back-propagated from the Softmax layer to the convolutional layer. The calculation formula is as follows:

(23) $$L_{c}=-\sum_{i=1}^{K}y_{i}\mathrm{lg}p_{i}$$

where $L_{c}$ is the cross-entropy loss function, K is the total number of categories, and $p_{i}$ is the probability that the target predicted by the network belongs to the category *i*. When the cross-entropy loss function is lower, it indicates that the model’s predicted results are closer to the actual outcomes, resulting in improved effectiveness in model training.

**Figure 15:**
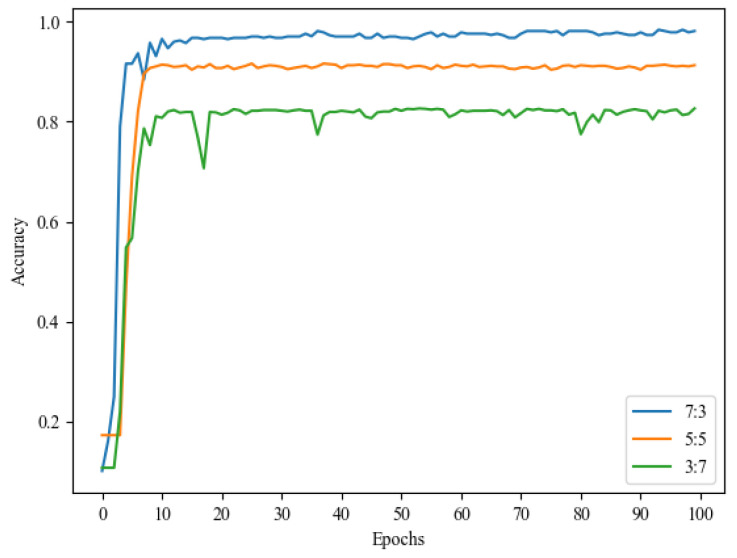
Accuracy of scale models on different datasets.

To evaluate the impact of training set size on model effectiveness, this study experimented with different ratios of test dataset sizes: 3:7, 5:5, and 7:3. Figure 15 illustrates the accuracy results over time. As shown in the figure, it becomes evident that the model’s performance deteriorates when the training set size is smaller. An insufficient amount of data limits the model’s learning capacity. However, when the proportion of the training data increases to 70% (7:3 ratio of training set to test set), the model demonstrates stronger generalization ability and achieves significantly higher accuracy. After the model was trained for about 15 epochs, the accuracy tended to stabilize, which indicates that the model converges easily. Based on this finding, this study selected a training set-to-test set ratio of 7:3 for further experiments.

This paper evaluates the performance of various models on the micro-Doppler spectrum test set using accuracy, recall, F1 score, parameter count, and memory usage as evaluation metrics.

### 4.3. Model Performance Analysis

**Figure 16:**
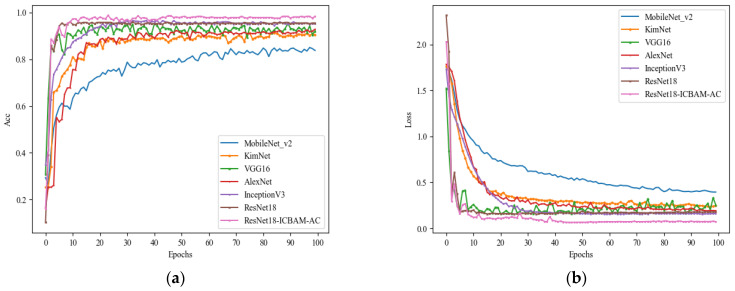
(**a**) Accuracy curves of different models. (**b**) Loss value curves of different models.

To validate the efficacy of the proposed method, this paper experimentally compares the ResNet18-ICBMA-AC model to other classic deep learning models, including MobileNet_V2 [27], VGG16 [28], AlexNet [29], InceptionV3 [30], ResNet18, and the KimNet network model cited in the literature [16]. The experimental comparison results are presented in Table 2. Additionally, the comparison of experimental accuracy and loss values for different models is shown in Figure 16.

As illustrated in Table 2, although the memory size of the ResNet18-ICBMA-AC model proposed in this article is slightly higher than that of the original ResNet18, its accuracy, recall, and F1 score have been significantly improved. The average accuracy reached 98.28%, which is 3.23 percentage points higher than that of ResNet18.

Compared to classic networks such as VGG16, AlexNet, KimNet, and InceptionV3, these models not only exhibit relatively high parameter counts and memory requirements but also achieve lower accuracy than the network model proposed in this paper. This limitation restricts their deployment on mobile devices or memory-constrained environments. For instance, VGG16 achieved an accuracy of only 91.4%. This comparison underscores that increasing network depth by adding layers with smaller convolution kernels and pooling may not be an effective strategy for micro-Doppler spectrum action recognition tasks.

In particular, when dealing with small micro-Doppler spectrogram datasets, there is a heightened risk of overfitting, leading to poor performance on unseen data. Additionally, while MobileNet_V2 boasts a smaller memory footprint, its performance ranks the lowest among all the network models evaluated. This highlights the importance of striking a balance between performance metrics and resource consumption when selecting a model.

**Figure 17:**
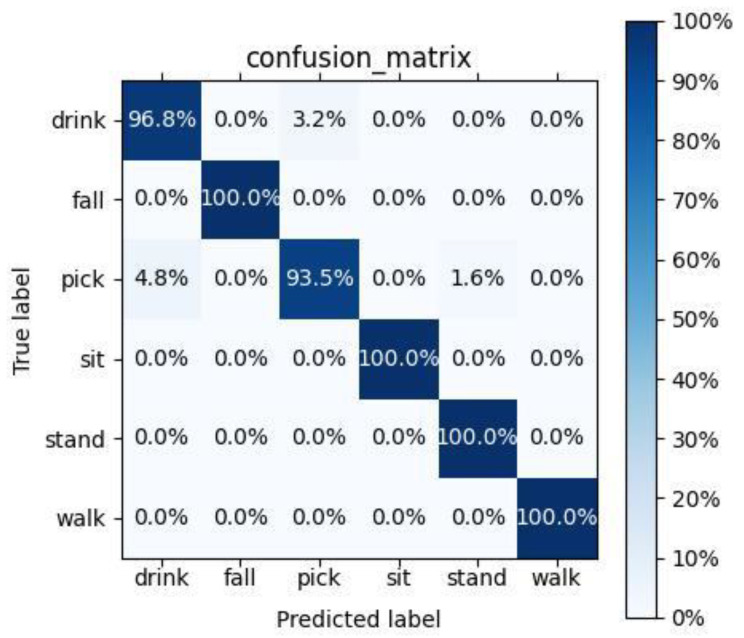
Confusion matrix.

Figure 17 displays the confusion matrix of the ResNet18-ICBMA-AC network model proposed in this paper for classifying six different actions. The horizontal axis represents the predicted action labels by the model, while the vertical axis represents the actual action labels. The diagonal line indicates the accuracy of correct classifications by the model.

From the figure, it is evident that the model achieved 100% recognition accuracy for the actions of walking, falling, standing up, and sitting down. However, there were some misclassifications in identifying the actions of picking up objects and drinking from a cup. Overall, the average classification accuracy of the improved model reached approximately 98.28%. When distinguishing actions with similar micro-Doppler characteristics, the minimum recognition accuracy was around 93.5%.

### 4.4. Ablation Experiment

In this paper, several experimental schemes are designed for ablation experiments to verify the effectiveness of the proposed method. Specifically, these methods include adjusting the convolution kernel size in the Stem module, introducing the ICBAM attention mechanism in the residual block, and employing asymmetric convolution. The experimental settings are detailed in Table 3, with Scheme 5 representing the ResNet18-ICBAM-AC network structure used in this paper.

Through these experiments, this paper systematically evaluates the contribution of each improved module to the overall model performance.

The results of the modeling tests are presented in Table 4. Comparing the results of Scheme 1 and Scheme 2, it is shown that replacing the convolutional kernel in the Stem module improves the accuracy by 0.67%. This improvement is due to the use of three cascaded 3 × 3 convolution kernels to enhance the depth of the network, enabling more effective learning of multi-level feature representations from the input data.

Comparing Scheme 2 and Scheme 3, it is observed that replacing the original convolution block with fused asymmetric convolution improves the model’s recognition accuracy by 1.13%. Specifically, the accuracy in recognizing drinking actions significantly improved, and the accuracy for standing actions reached 100%. This enhancement underscores the effectiveness of fused asymmetric convolution.

By employing convolution kernels of different sizes, the model can more fully utilize local information from the input data, thereby improving its ability to extract detailed features from the spectrogram. This approach also enhances the model’s adaptability to diverse data types. Additionally, the introduction of the Mish activation function increases the model’s nonlinearity, aiding in mitigating issues like gradient explosion and facilitating faster convergence towards a globally optimal solution.

Comparing Scheme 3 and Scheme 5, the introduction of the ICBAM improves the model’s accuracy by 1.33%, while the model’s memory usage does not increase significantly. Additionally, this paper conducted comparative experiments on different attention mechanisms, as shown in Table 5. The results demonstrate the superiority of the attention mechanism proposed in this paper over other methods.

**Figure 18:**
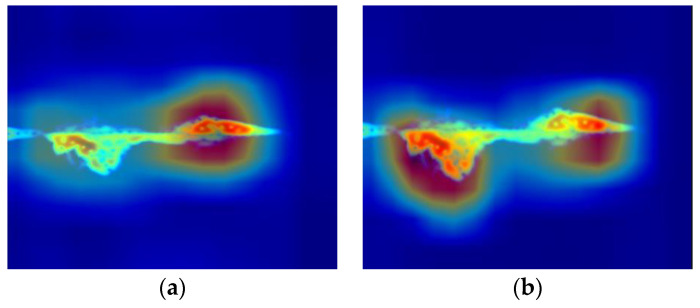
Attention heat map: (**a**) model without ICBAM and (**b**) model with ICBAM.

Furthermore, this paper utilizes Grad-CAM [31] to visualize the network and observe the areas of focus during the decision-making process. This visualization helps verify the contribution of the ICBAM attention mechanism in enhancing network performance. As depicted in the attention heat map in Figure 18 for the micro-Doppler spectrum map depicting the action of picking up an object, the red area highlights where the network focuses. It is evident that with the introduction of the ICBAM, the model effectively captures and utilizes crucial information from the spectrum map while reducing the influence of background noise, thereby significantly improving the recognition accuracy.

**Figure 19:**
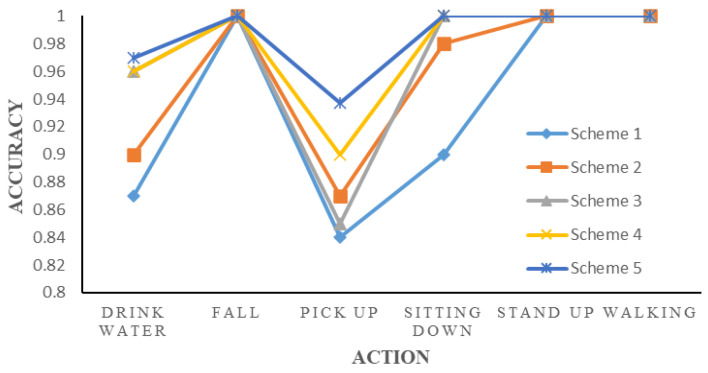
Action accuracy of each scheme.

In order to clearly demonstrate the improvements made by this model for micro-Doppler spectrum action recognition, Figure 19 displays the accuracy results for each action based on the confusion matrix. This article lists the accuracy of each action recognition tested by different schemes. It is evident from the results that the optimization scheme not only enhances the recognition accuracy of different actions but also enables more accurate differentiation among activities that exhibit similar Doppler characteristics. This capability enhances the model’s applicability in real-world environments.

### 4.5. Noise Immunity Performance Analysis

**Figure 20:**
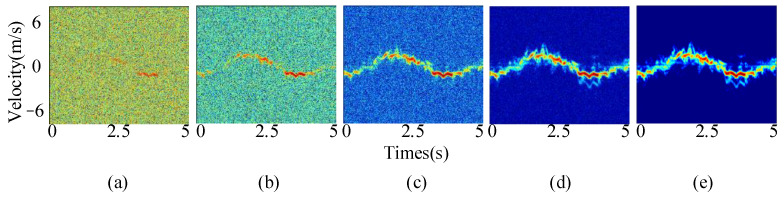
Spectra with different signal-to-noise ratios: (**a**) −20 dB; (**b**) −10 dB; (**c**) 0 dB; (**d**) 10 dB; and (**e**) 20 dB.

To test the noise immunity of the model, Gaussian white noise with varying signal-to-noise ratios was introduced into the micro-Doppler spectrum. As depicted in Figure 20, this was employed to evaluate the model’s capability to recognize different noise spectra.

Table 6 shows that the model maintains an accuracy of 94.84% even when the signal-to-noise ratio decreases to −10 dB. However, as the signal-to-noise ratio decreases further, the noise begins to dominate the spectrogram features, causing the model to struggle to converge. This demonstrates the excellent discrimination and robust noise immunity of the proposed model when dealing with micro-Doppler spectrograms with signal-to-noise ratios higher than −10 dB.

### 4.6. Performance Comparison of Different Radar Recognition Methods

Table 7 presents the results of other studies on human action recognition, comparing their accuracy to that of the method proposed in this paper.

In reference [32], the same dataset that was analyzed in this study was utilized to classify human actions using two machine learning methods: SVM and K-Nearest Neighbors (KNN). The SVM classifier achieved an accuracy of 78.25%, while the KNN classifier achieved an accuracy of 77.15%.

Reference [33] uses grayscale spectrograms as the network input, extracts feature through stacked autoencoders, and, finally, uses a logistic regression classifier for activity classification. This method achieved an accuracy of 89.4% in the experiment.

Some authors combined the features of the time–range domain spectrum, range–Doppler domain spectrum, and time–Doppler domain spectrum and used the CNN–LSTM combined network as the classification model, achieving an accuracy of 94.25% [19].

This paper uses the micro-Doppler spectrogram as the input feature and ResNet18-ICBAM-AC as the classification model to further improve the classification accuracy. Additionally, it utilizes real indoor and outdoor datasets, covering various venues and involving multiple people. This demonstrates that our method exhibits high performance and robustness in human action recognition, outperforming other methods.

## 5. Conclusions

This paper introduces a novel method for human action recognition based on micro-Doppler characteristics applied to an FMCW radar system. The method extracts subtle changes in various actions from radar echo signals and translates them into radar signal spectra in the time domain. These micro-Doppler images are then classified using the ResNet18-ICBAM-AC network architecture proposed in this study. By enhancing the convolutional structure of the Stem module, introducing the ICBAM attention mechanism, and employing a strategy that combines asymmetric convolution with the Mish activation function, an average classification accuracy of 98.28% is achieved. Even when dealing with micro-Doppler spectra that have similar features or high noise levels, this method maintains high classification accuracy, highlighting its effectiveness in human action recognition based on FMCW radar.

Since this system is not integrated into the embedded system, it still needs to be operated and displayed manually on the computer side, which may lead to delays and affect the real-time performance of the system. In future work, this study will integrate signal processing and activity recognition networks into embedded systems, ensuring further miniaturization and real-time performance of human activity detection systems through operations such as model pruning.

## Figures and Tables

**Table 1:** Experimental environment parameters.

Experimental Environment	Parameters
Operating system	Linux
Graphics card	NVIDIA GeForce RTX 3080
Deep learning frameworks	PyTorch
Batch size	64
Epoch	100
Optimizer	Adam
Initial learning rate	0.0001
Input image size	224 × 224

**Table 2:** Comparison of the results of different models.

Model	Accuracy/%	Recall/%	F1 Score/%	Parameter Quantity/10^7^	Memory Size/MB
MobileNet_V2	83.80	83.96	83.87	0.22	8.5
KimNet	89.11	89.37	89.23	1.66	63.4
VGG16	91.40	91.78	91.55	13.4	512.3
AlexNet	92.55	93.01	92.80	5.70	217.6
InceptionV3	94.84	95.16	95.14	2.18	83.5
ResNet18	95.05	95.16	95.09	1.12	42.7
ResNet18-ICBMA-AC	98.28	98.39	98.38	1.13	43.2

**Table 3:** Experimental design.

Scheme Number	Stem Module		ICBAM		Residual Block	
	7 × 7	3 × 3 × 3	Not Use	Use	Ordinary Convolution	Fused Asymmetric Convolution
1	√		√		√	
2		√	√		√	
3		√	√			√
4	√			√		√
5		√		√		√

**Table 4:** Test results of each scheme.

Scheme Number	Accuracy/%	Recall/%	F1 Score/%	Memory Size/MB
1	95.05	95.16	95.09	42.7
2	95.72	95.97	95.87	42.9
3	96.85	97.04	97.05	43.2
4	97.42	97.61	97.58	43.0
5	98.28	98.39	98.38	43.2

**Table 5:** Comparison of attention mechanisms.

Model	Accuracy/%	Parameter Quantity/10^7^	Memory Size/MB
ResNet18	95.05	1.12	42.7
ResNet18 + SENet	95.53	1.13	43.0
ResNet18 + CBAM	96.19	1.13	43.3
ResNet18 + ICBAM	96.62	1.12	42.7

**Table 6:** Recognition accuracy of graphs with different signal-to-noise ratios.

Signal-to-Noise Ratio	20 dB	10 dB	0 dB	−10 dB	−20 dB
Accuracy (%)	98.28	97.67	96.53	94.84	--

**Table 7:** Comparison of the network performance of different studies.

Input Features	Action Type	Number of Experimenters	Network Structure	Accuracy %
Micro-Doppler spectrogram [32]	6	56	KNN	77.15
Micro-Doppler spectrogram [32]	6	56	SVM	78.25
Denoised spectrogram grayscale image [33]	4	-	Stacked Autoencoders	89.40
Time–Range, Time–Doppler, and Range–Doppler three-domain fusion [19]	6	56	CNN+LSTM	94.25
Range–Doppler map [34]	7	20	CNN	95.00
Micro-Doppler spectrogram	6	56	ResNet18-ICBMA-AC	98.28

## Data Availability

The data are unavailable due to the privacy policy.
